# Fundamental electronic changes upon intersystem crossing in large aromatic photosensitizers: free base 5,10,15,20-tetrakis(4-carboxylatophenyl)porphyrin†

**DOI:** 10.1039/d1cp05420a

**Published:** 2022-03-15

**Authors:** Robby Büchner, Vinícius Vaz da Cruz, Nitika Grover, Asterios Charisiadis, Mattis Fondell, Robert Haverkamp, Mathias O. Senge, Alexander Föhlisch

**Affiliations:** Institute of Physics and Astronomy, University of Potsdam Karl-Liebknecht-Str. 24-25 14476 Potsdam Germany rbuechner@uni-potsdam.de; Institute for Methods and Instrumentation for Synchrotron Radiation Research, Helmholtz-Zentrum Berlin für Materialien und Energie Albert-Einstein-Str. 15 12489 Berlin Germany vinicius.vaz_da_cruz@helmholtz-berlin.de; School of Chemistry, Chair of Organic Chemistry, Trinity College Dublin, The University of Dublin, Trinity Biomedical Sciences Institute 152-160 Pearse Street Dublin 2 Ireland; Institute for Advanced Study, Technical University of Munich Lichtenbergstrasse 2a 85748 Munchen Garching Germany mathias.senge@tum.de

## Abstract

Free base 5,10,15,20-tetrakis(4-carboxylatophenyl)porphyrin stands for the class of powerful porphyrin photosensitizers for singlet oxygen generation and light-harvesting. The atomic level selectivity of dynamic UV pump – N K-edge probe X-ray absorption spectroscopy in combination with time-dependent density functional theory (TD-DFT) gives direct access to the crucial excited molecular states within the unusual relaxation pathway. The efficient intersystem crossing, that is El-Sayed forbidden and not facilitated by a heavy atom is confirmed to be the result of the long singlet excited state lifetime (Q_*x*_ 4.9 ns) and thermal effects. Overall, the interplay of stabilization by conservation of angular momenta and vibronic relaxation drive the de-excitation in these chromophores.

## Abstract 2

The crucial transient states of free-base porphyrins are characterized by time-resolved X-ray absorption spectroscopy unraveling their unusual relaxation pathway.

## Graphical abstract

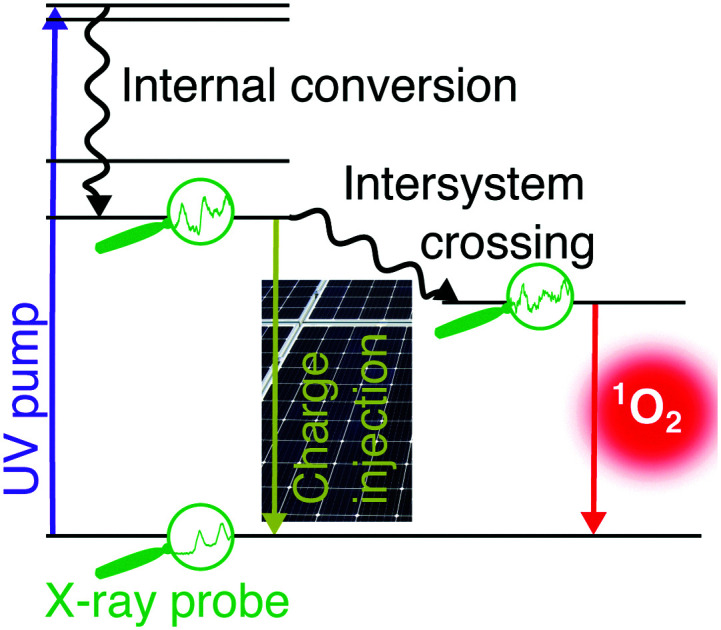

## 1 Introduction

Apart from the potential in future photovoltaics,^1^ free base porphyrins are efficient photosensitizers for the generation of singlet oxygen – a highly reactive oxidizing agent.^2^ As a consequence, the accumulation of free base porphyrins in plants and vertebrates such as humans leads to pathological photosensitivity.^3–5^ On the other hand, the high singlet oxygen yield of free base porphyrins in a wide spectral range is employed in the treatment of tumors,^6,7^ atherosclerosis,^8^ skin diseases,^9^ and microbia^10^ by photodynamic therapy (PDT), for sustainable chemistry,^11,12^ and photocatalysis.^13,14^

**Fig. 1:**
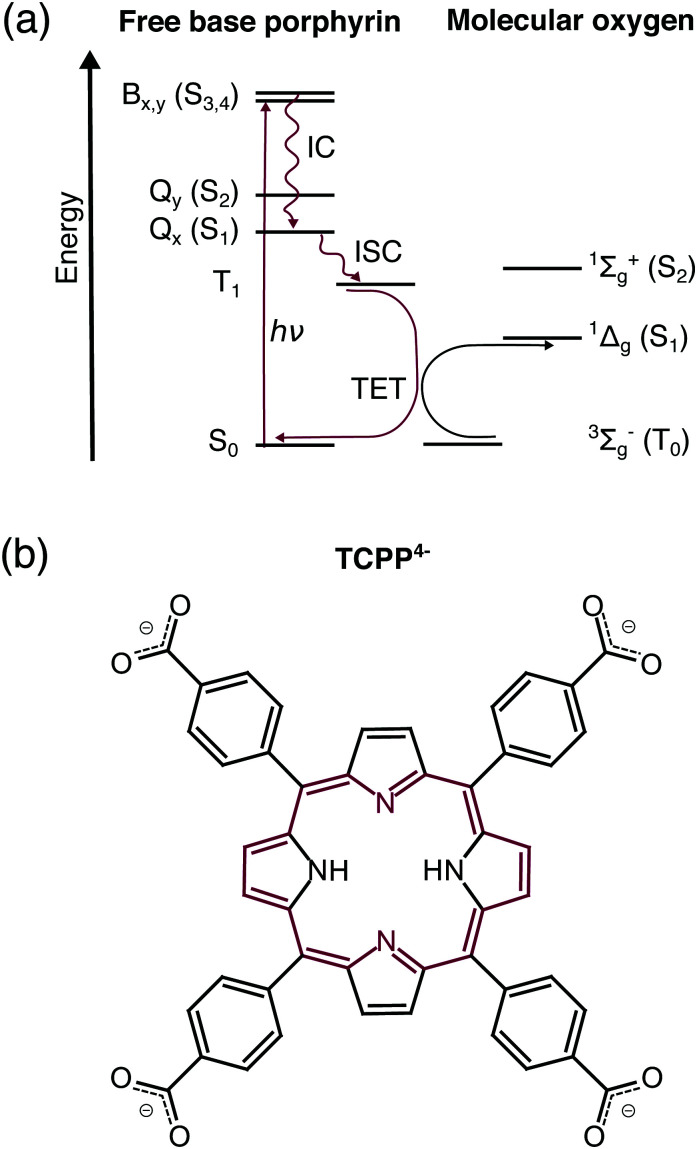
(a) Dominant relaxation pathway of optically exited free base porphyrins in the presence of molecular oxygen: IC – internal conversion, ISC – intersystem crossing, TET – triplet energy transfer. (b) Structural formula of free base 5,10,15,20-tetrakis(4-carboxylatophenyl)porphyrin (TCPP^4−^). The 18 π-electron aromatic system is highlighted in red.

In all these cases, free base porphyrins are excited by ultraviolet or visible light (UV/VIS) to one of the singlet excited states (Fig. 1a). Higher excited states (Q_*y*_, B) are transformed to the lowest singlet excited state (Q_*x*_) by ultrafast internal conversion.^15^ The nanosecond lifetime of Q_*x*_ in combination with vibronic coupling were predicted to facilitate the efficient intersystem crossing to the lowest triplet state (T_1_).^9,16^ In the presence of oxygen, triplet free base porphyrin decays to the singlet ground state (S_0_) by triplet energy transfer raising ground state oxygen (^3^Σ_g_^−^ O_2_) to its first singlet excited state (^1^Δ_g_ O_2_).^17^ While there is a general agreement in the literature about this abstract deactivation path, the exact electronic structure of the involved states is debated especially regarding the energetic order of the frontier orbitals in T_1_.^18^

Free base 5,10,15,20-tetrakis(4-carboxylatophenyl)porphyrin (TCPP^4−^, Fig. 1b) is the parent compound of novel agents for photodynamic diagnosis and PDT of breast and skin cancer with singlet oxygen quantum yields up to *Φ*_*Δ*_ = 0.61.^19^ Compared to previous studies on lipophilic porphyrins, such as free base 5,10,15,20-tetraphenylporphyrin (TPP), this water-soluble porphyrin allows the investigation in aqueous solution mimicking the water-containing environment in a biological cell.^20^ Considering the light-harvesting applications, carboxylate moieties of TCPP^4−^ are typical anchoring groups in dye-sensitized solar cells.^14^ Therefore this molecule is an ideal candidate for transient electronic structure investigation of solar cell chromophores subsequent to the existing work on zinc porphyrins.^21^

In this work, we monitor the relaxation of photoexcited TCPP^4−^ on an atomic level with focus on the configurations and lifetimes of the long-lived lowest singlet and triplet excited states. Therefore UV pump – N K-edge probe spectroscopy is employed yielding the evolution of the near-edge X-ray absorption fine structure (NEXAFS) after the photoexcitation. The spectra are interpreted with the aid of TD-DFT calculations within the restricted subspace approximation (RSA)^22^ providing detailed information on the electronic structure before and after the intersystem crossing, as well as evidence for the theoretically proposed vibronic deactivation channels.

## 2 Methods

The precursor 5,10,15,20-tetrakis(4-methoxycarbonylphenyl)porphyrin (TCOOMePP) and desired TCPP compounds were synthesized following previously reported procedures^23,24^ (see Synthesis for details, ESI†). The final 3 mM TCPP^4−^ solution (pH ≈ 12) was prepared with deionized water and NaOH. The solute is expected to be fourfold deprotonated since all carboxyl groups independently deprotonate with p*K*_a_ ≈ 6.^25^

Preparatory measurements were carried out at beamline UE49-SGM^26^ with the EDAX endstation^27^ (Bessy II, Berlin). The static and transient data has been acquired with the nmTransmission NEXAFS^28^ endstation at UE52-SGM.^29^ In this setup a thin leaf, that is formed upon the collision of two liquid jets, is used to directly determine the X-ray transmission of the sample solution. The liquid jets enter the vacuum chamber *via* a pair of 30 μm sized nozzles with a combined flow rate of 1.4 mL min^−1^.

The sample was excited at 343 nm with a pulse energy of 7 μJ and a spot size of (80 × 80) μm^2^. A repetition rate of 208 kHz was chosen, to allow full sample replenishment between the UV pulses. The X-ray probe had a bandwidth of 0.13 eV and spot size of (55 × 140) μm^2^. The temporal resolution of the experiment is limited by the length and jitter of the synchrotron bunches and amounts to 0.14 ± 0.01 ns according to the fit of the delay traces. The static, 0.1 ns, 5.0 ns, and 40.0 ns delayed transient spectra were in total acquired for 30 s, 13 s, 6 s, and 3 s per 0.05 eV step, respectively. Keeping the X-ray photon energy fixed and varying the pump–probe delay from −0.5 ns to 1.0 ns and 1.0 ns to 40.0 ns yielded the time traces, each with 61 steps and a net acquisition time of 11 minutes.

All photon energies were calibrated by the signature of co-dissolved N_2_ in the ground state spectrum.^30,31^ The shown static spectrum was yielded by subtracting the fitted N_2_ signature and solvent background.

For the theoretical description, the parent carboxylate-free TPP was considered. The influence of the weakly electron donating carboxylate groups for the probe of the local electronic structure at the nitrogen sites is expected to be small. This assumption is based on the high similarity in the experimental N K-edge spectra of TCPP and TPP.^32,33^ The ORCA package^34^ was used for all electronic structure calculations. The aqueous environment of the experimentally investigated molecules was modeled by the conductor-like polarizable continuum model (CPCM).^35^ The B3LYP^36,37^ functional was used with the def2-TZVP(-f)^38^ basis set, def2/J^39^ auxiliary basis set, and Becke–Johnson damping.^40,41^ The choice of these parameters is based on our past benchmark^32^ and the computational efficiency needed for the simulation of multiple core- and valence-excited states. The geometry optimization was carried out for the S_0_, T_1_, and Q_*x*_ state without symmetry restrictions to yield more accurate geometries regarding the tilt of the phenyl groups^32^ and deformations of the porphyrin macrocycle in the excited states. The given configuration interaction coefficients are the result of ground state TD-DFT calculations.

To compute the transient signals, we employed the restricted subspace approximation^22^ in the TD-DFT spectrum calculations using Multiwfn^42^ to compute the transition dipole moments between the involved states (see Application of the restricted subspace approximation for details, ESI†). The lowest excitations from the localized –N= and –NH– 1s orbitals have been determined for the minimum geometry of the respective electronic state. The resulting spectra were shifted by 12.5 eV and broadened by 0.13 eV (Gaussian FWHM) and 0.5 eV (Lorentzian FWHM^43^) according to the lowest experimental ground state transition.

## 3 Results and discussion

**Fig. 2:**
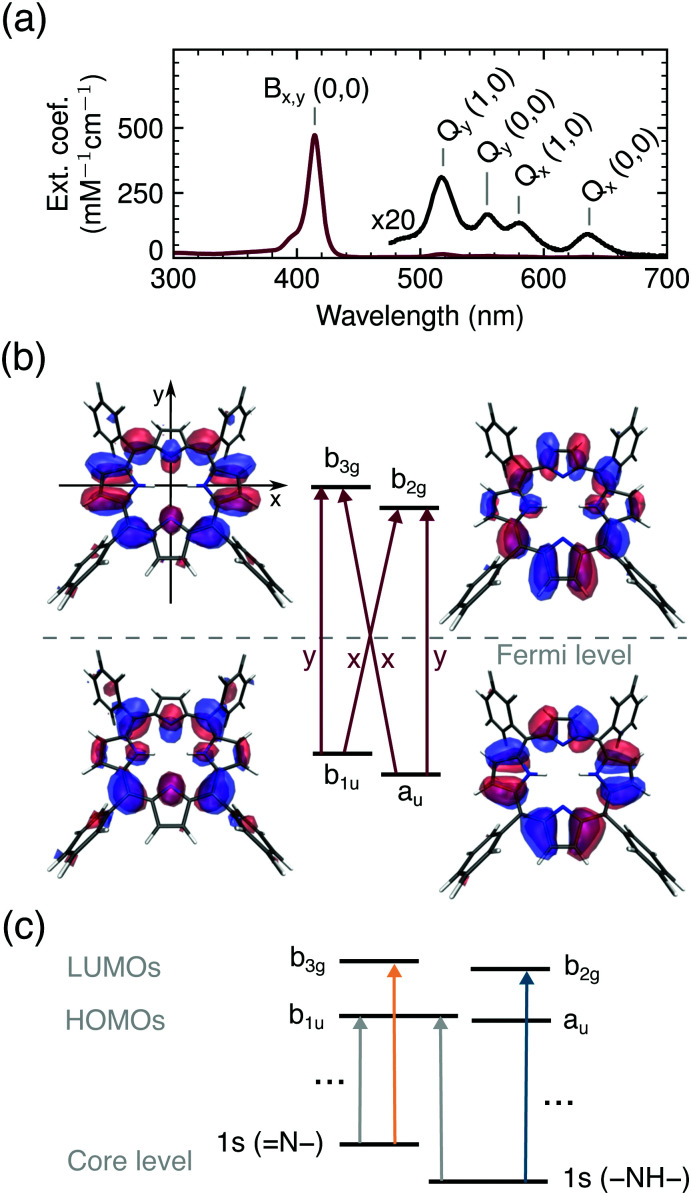
(a) UV/VIS spectrum of TCPP^4−^. (b) Frontier orbitals with the porphyrin macrocycle in the *xy*-plane and polarization of optical transitions in red. (c) Schematic representation of X-ray induced transitions.

The first model of the porphyrin electronic structure, that successfully explains the UV/VIS spectra (Fig. 2a), was proposed by Gouterman in 1959^44,45^ and is used to the present day. According to this model and our calculations, all bands in the optical spectrum of TCPP^4−^ (Fig. 2a) are related to transitions between the two highest occupied (HOMOs) and lowest unoccupied (LUMOs) molecular orbitals and hence of π → π* character. We use the irreducible representations to describe these orbitals throughout this work to account for the *D*_2h_ symmetry of the free base porphyrin macrocycle (oriented as shown in Fig. 2b). From group theory, it can be deduced that the optical transitions are either *x* (b_1u_ → b_2g_, a_u_ → b_3g_) or *y* (b_1u_ → b_3g_, a_u_ → b_2g_) polarized, as shown in Fig. 2b. Transitions of the same polarization (*x* or *y*) are expected to mix according to their proximity in energy.

While the two lowest unoccupied orbitals are degenerate in metalloporphyrins (where the central protons are replaced by a divalent metal ion) the b_2g_ orbital is lowered in energy for ground state free base porphyrins due to the electron density on the aminic nitrogen atoms.^46^ The order of the HOMOs depends on the peripheral substituents. If they are linked to the bridging (*meso*) carbon atoms, the b_1u_ orbital is slightly higher in energy than the a_u_ orbital^47^ (see Fig. 2b).

Even though, the frontier orbitals are not completely pairwise degenerate, the aromatic porphyrin macrocycle can be approximated by a free electron ring^48^ to explain the absorption spectrum (Fig. 2a). From the nodes of the wavefunctions (depicted in Fig. 2b) the orbital angular momentum normal to the porphyrin plane can be derived: *l*^HOMOs^_*z*_ ≈ ±4*ħ* and *l*^LUMOs^_*z*_ ≈ ±5*ħ*. According to the ground state of total angular momentum 
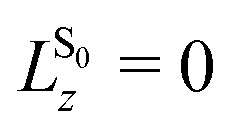
 and the selection rule Δ*L*_*z*_ = ±1*ħ*, transitions to the B_*x*_/B_*y*_ state (*L*^B^_*z*_ ≈ ±1*ħ*) are high in energy and allowed, while the opposite applies to transitions to the Q_*x*_/Q_*y*_ states (*L*^Q^_*z*_ ≈ ±9*ħ*).^48^ The quasi-forbidden character of the Q_*x*_/Q_*y*_ transition can be lifted by in-plane deformations, giving rise to the lower intensity of Q_*x*_(0, 0)/Q_*y*_(0, 0) compared to Q_*x*_(1, 0)/Q_*y*_(1, 0) denoting transitions to vibrationally excited modes.^9,49^ If the near-degeneracy of B_*x*_ and B_*y*_ is considered, the UV/VIS spectrum of TCPP^4−^ is fully understood.

**Fig. 3:**
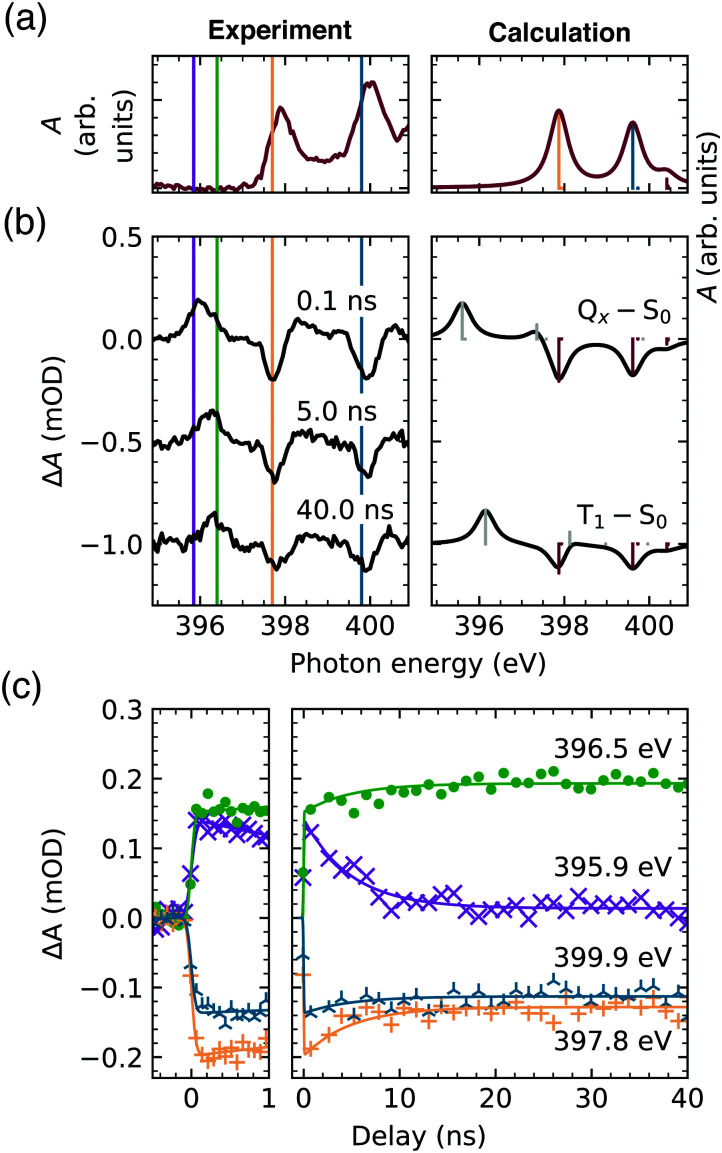
(a) Ground state TCPP^4−^ N K-edge NEXAFS spectrum. (b) Transient spectra 0.1 ns, 5.0 ns and 40.0 ns after the laser excitation (left) in comparison to the calculated spectra (right). (c) Experimental time traces at the resonance energies.

With X-ray absorption spectroscopy, we are able to probe the electronic structure with atomic precision, enabling a detailed picture of energies and occupancies of the TCPP^4−^ frontier orbitals (Fig. 2c). Opposed to the near-degenerate HOMOs/LUMOs, the aminic and iminic pairs of nitrogen core levels are shifted by as much as 2 eV,^50,51^ since the higher electron density at the iminic nitrogens screens the core charge more efficiently.^52^ Consequently, the energetically lowest resonance (397.9 eV) in the experimental and calculated ground state N K-edge NEXAFS (Fig. 3a) is of 1s(=N–) → π* character. Since only the b_3g_ unoccupied orbital has amplitude at the iminic nitrogens, it is populated by the core electron in this transition. For the same reasons, the 400.0 eV resonance corresponds to the 1s(–NH–) → b_2g_ transition. At higher excitation energies less prominent features with only small transient changes are observed (see full spectrum in Fig. S1, ESI†). A detailed interpretation of the ground state spectrum is given elsewhere.^32^

The first TCPP^4−^ transient signal that is probed with the temporal resolution of our setup (0.1 ns in Fig. 3b) is expected to probe the lowest singlet excited state (Q_*x*_). From the parent TPP it is known, that this state is electronically populated and thermally equilibrated in less than 100 fs and 20 ps, respectively, after optical or UV excitation under ambient conditions.^15^ The π → π* transition, leading to the Q_*x*_, opens a new channel for the core excitation, *viz.* the 1s(N) → b_1u_ transition, which is equally probable for both iminic and aminic nitrogen sites (Fig. 2c). The former lead to a new feature below the edge of the ground state (396.0 eV). The second feature, corresponding to 1s(–NH–) → b_1u_ transitions, overlaps with other transient features (gray bar around 398 eV in Fig. 3b). Potential transitions to a_u_ are not observed, as this π orbital does not have any amplitude at any nitrogen site. The depletion of the ground state (red bars in Fig. 3b) gives rise to the remaining strong transient features.

For increasing delay times of the X-ray probe to the UV excitation (5.0 ns and 40.0 ns in Fig. 3b) the 1s(=N–) → b_1u_ transient feature shifts to higher energies (396.4 eV). This shift is reproduced by the calculations when comparing the lowest singlet (Q_*x*_–S_0_) and triplet transient (T_1_–S_0_) evidencing the direct observation of an intersystem crossing.

Fig. 3c shows the continuous temporal evolution of the transient features until 40 ns after laser excitation. The time traces were fitted by an exponentially modified Gaussian distribution (with an identical lifetime) and a step function being convoluted with the same Gaussian broadening, since a second, slower decay cannot be unambiguously identified within the 40 ns time window. The short-lived component is most prominent in the time evolution of the 396.0 eV feature (probed by the time trace at 395.9 eV, magenta), verifying that this feature is the signature of the lowest singlet excited state. The equivalent feature of the triplet state (probed at 396.5 eV, green) shows a clear delay of the initial increase in absorbance even though it energetically overlaps with the just discussed peak at 396.0 eV. In the case of the two depletions, of which the temporal evolution has been captured at 397.8 eV (orange) and 399.9 eV (blue), the reduction of the absorbance compared to the ground state is observed both in the lowest singlet and triplet excited state.

The global fit of the singlet lifetime yields *τ*_F_ = 4.9 ± 0.5 ns, which is in the range of known TCPP fluorescence lifetimes, *i.e.* from 4.0 ns in organic solvents to 10.4 ns in basic aqueous solution.^19,20,25,53^ Our result rather corresponds to the lifetimes in less polar solutions agreeing with the negligible influence of the solvent on the Q_*x*_ lifetime, which has recently been established in a review of TPP (and ZnTPP) photophysical properties.^54^ Instead, the O_2_ saturation of the solution has been considered as the dominant factor – leading to a 23% decrease – of the lifetime of the lowest singlet excited state. However, one of the shorter TCPP lifetimes^19^ has been determined in de-aerated solutions, while one of the longer ones in air-equilibrated solution.^53^ Also, the influence of aggregation on the Q_*x*_ lifetime in our concentrated aqueous solution can be excluded, as TCPP aggregates show fluorescence lifetimes below 1 ns.^53^ The large variation indicates that temperature should be considered as the main parameter determining the singlet state lifetime. This supports the proposed vibronic nature of the intersystem crossing in free base porphyrins.^9^

**Fig. 4:**
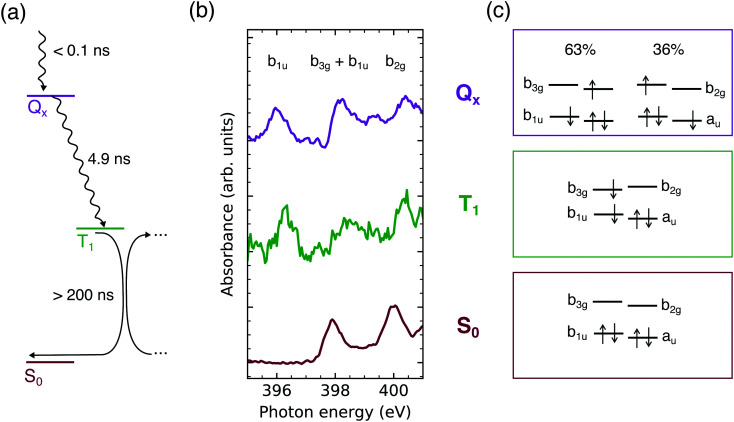
(a) Jablonski diagram of the TCPP^4−^ deactivation pathway in the nanosecond regime. (b) Approximated X-ray absorption spectra probing the TCPP^4−^ frontier orbital occupation in the lowest singlet excited (Q_*x*_), triplet (T_1_), and ground state (S_0_). (c) Calculated configurations in these states.

From the time traces, a lower limit for the lifetime of the triplet state can be inferred: *τ*_T_ > 200 ns. This agrees with previously observed triplet state lifetimes of *τ*_T_ > 1 μs dependent on the oxygen concentration^11,15,55^ and potential triplet–triplet annihilation in concentrated solutions.^56^ All determined lifetimes are summarized in Fig. 4a.

For closer analysis of the electronic structure in the two observed excited states, the Q_*x*_ and T_1_ absorption spectra have been approximated by adding the S_0_ spectrum to the transient ones so that the ground state depletion is compensated (see Fig. S2, ESI†). The resulting spectra should be viewed with caution as the spectral intensities depend on Franck–Condon progressions^57–59^ and the exact fraction of excited molecules. However, the results gained by this naive approach (Fig. 4b) agree with the calculated electronic configurations (Fig. 4c) and are therefore used as illustration.

From the earlier discussion on the UV/VIS spectrum, the calculated mixture of ^1^(b_1u_ b_2g_) and ^1^(a_u_ b_3g_) configurations in the Q_*x*_ state is expected. The dominance of the former by 27% (Fig. 4c) is supported by the Q_*x*_ absorbance in the 1s(N) → b_3g_/b_1u_ and 1s(N) → b_2g_ energy regions compared to the other states (Fig. 4b). The deviation from the 50 : 50 mixture, being expected in an idealized free electron ring, is a result of the deviations from the pairwise degeneracy of the frontier orbitals.^46^ However, as seen from the UV/VIS spectrum, electric dipole transitions between the ground and lowest singlet excited state are still quasi-forbidden. Therefore also fluorescence from this state is unfavorable with a fluorescence quantum yield *Φ*_F_ ≤ 0.25^11,19,20,25^ as supported by the similar intensity of our transient spectra at short and long delays (Fig. 3b and c).

The long lifetime of the singlet state gives rise to the high triplet yield (*Φ*_T_ = 0.78^11^) despite the lack of both a heavy atom and close-lying non-ππ* intermediate states which would facilitate the process according to El-Sayed's rule.^9,60^ Instead, a crossing of the Q_*x*_ and T_1,2_ potential energy surfaces along the central proton transfer reaction path was proposed^16^ and experimental evidence for the tautomerism has recently been found.^61^ Since the spin–orbit coupling matrix element increases to an amount that makes the transition competitive with fluorescence only for out-of plane distortions, thermal activation and a long-lived singlet state are prerequisites for the intersystem crossing.

An intermediate, higher triplet state as a result of the intersystem crossing^16^ is not visible within our temporal resolution. Instead, a pure one electron excited state is obtained, as mixing with other configurations is restricted by symmetry in the lowest triplet states.^48,62^

The approximated T_1_ spectrum suggests a ^3^(b_1u_ b_3g_) configuration. This is in agreement with studies on TPP^47^ and free base 5,10,15,20-tetrakis(4-sulfonatophenyl)porphyrin.^61^ For TCPP^4−^ a contraction of the solvation shell upon triplet state formation has been observed,^25^ which can be explained by the charge density difference of ^3^(b_1u_ b_3g_) compared to the ground state: b_1u_ (fully occupied in S_0_) has equal amplitudes at all nitrogen sites, but b_3g_ (unoccupied in S_0_) only at the iminic ones (Fig. 2b). When both molecular orbitals are singly occupied, the charge at the aminic sites decreases. As this strengthens the hydrogen bond between water and the amino group (H_2_O⋯HN^*δ*+^), the solvation shell is contracted in the lowest triplet state. This supports the presence of the ^3^(b_1u_ b_3g_) configuration for TCPP^4−^.

It should be noted that the triplet state in non-*meso*-substituted porphyrins is expected to be of ^3^(a_u_ b_3g_) character, due to the different order of the HOMOs.^47,63,64^ In contrast, Kay proposed a ^3^(a_u_ b_3g_) configuration both for TPP and the parent, unsubstituted porphyrin.^18^ Since we only probe the b_1u_ orbital and detect an intense transient feature, this work provides experimental evidence, that the b_1u_ orbital is singly occupied in the lowest triplet state of TCPP^4−^, which also applies to TPP as shown by our calculations. These predict that the ^3^(b_1u_ b_3g_) state is energetically below ^3^(b_1u_ b_2g_) both in the ground state and triplet state geometry, but only in the latter one the order of the LUMOs is inverted.

A reduction of the free base porphyrin symmetry upon intersystem crossing^47^ and the resulting exchange of the LUMOs^18^ has been discussed in literature. However, out-of-plane distortions in the triplet state compared to the ground state as yielded by our calculations (see Fig. S3, ESI†) have rarely been addressed. Only recently, the importance of such distortions from the planar structure have been shown to be an essential factor for the triplet energy transfer to oxygen:^17^ while the porphyrin T_1_ vibrational ground state energy is close to the one needed for the excitation of ground state to singlet oxygen, the electron-exchange mechanism is most efficient if this deviation is minimized (resonance condition) by out-of-plane distortions.

In contrast, the electronic structure of the lowest singlet excited state is barley affected by small geometric distortions ensuring that fluorescence is quasi-forbidden. The resulting long lifetime is essential for the application in dye-sensitized solar cells. The charge injection into the conduction band of typically used semiconductors is energetically most favorable from the lowest singlet excited state. Since the decay of that state and charge injection are competing processes, a low decay rate is preferable.^65^ On the one hand, this competition can be steered by metal insertion leading to an increase of the electron injection rate. On the other hand, it has been shown that the main absorption band of free base porphyrins can be tuned to the “green gap” between the typical porphyrin absorption bands, to increase the overall efficiency of a solar cell.^1^ This feature might be of significant importance for future organic photovoltaics, where the energy of the triplet state can be harnessed.^66^ In that case – similar to the application as singlet oxygen photosensitizers – free base porphyrins are advantageous due to their long triplet state lifetimes.^67^

## 4 Conclusions

The relaxation of aqueous TCPP^4−^ after UV excitation has been observed by N K-edge NEXAFS spectroscopy. The lowest singlet excited state (Q_*x*_) is populated in less than 140 ps after the excitation. Despite of deviations from an ideal square planar porphyrin macrocycle, the 63 : 36 mixture of the ^1^(a_u_ b_3g_) and ^1^(b_1u_ b_2g_) configurations evidences that the free electron model is applicable. Therefore, fluorescence is forbidden by angular momentum conservation, which gives rise to the long lifetime of this state (4.9 ns).

In dye-sensitized solar cells, the low *Q*_*x*_ decay rate is a prerequisite for the electron injection. In isolated molecules, it enables the high yield of vibronic intersystem crossing by out-of-plane vibrational modes, whose thermal character has been confirmed by the variation of the *Q*_*x*_ lifetimes. The resulting long-lived triplet state (*τ*_T_ > 200 ns) is concomitant with a degree of structural bending. As a result the charge density is decreased at the aminic and increased at the iminic nitrogens. Further bending eases the triplet energy transfer to molecular oxygen. The resulting high quantum yield of this process is the basis for the various applications of free base porphyrins as photosensitizers for singlet oxygen generation.

## Author contributions

R. B.: data curation, investigation, project administration, visualization, original draft; V. V. C.: calculations; N. G. and A. C.: synthesis, M. F. and R. H.: investigation; M. O. S. and A. F.: conceptualization, funding acquisition, supervision.

## Conflicts of interest

There are no conflicts to declare.

## Supplementary Material

CP-024-D1CP05420A-s001
