# Supplementary material for: Fundamental electronic changes upon intersystem crossing in large aromatic photosensitizers: free base 5,10,15,20-tetrakis(4-carboxylatophenyl)porphyrin
Source: Phys Chem Chem Phys. 2022 Mar 15;24(12):7505–11. doi: 10.1039/d1cp05420a (PMC8942076; doi:10.1039/d1cp05420a)
Supplement: CP-024-D1CP05420A-s001 [file CP-024-D1CP05420A-s001.pdf]

Electronic Supplementary Material (ESI) for Physical Chemistry Chemical Physics.
This journal is © the Owner Societies 2022

Supplementary Information

Fundamental electronic changes upon intersystem crossing in large aromatic photosensitizers: free base 5,10,15,20-tetrakis(4-carboxylatophenyl)porphyrin

Robby Büchner*, Vinícius Vaz da Cruz*, Nitika Grover, Asterios Charisiadis, Mattis Fondell, Robert Haverkamp, Mathias O. Senge*, and Alexander Föhlisch

Contents

1 Synthesis **1**

2 Full ground state and transient spectra **1**

3 Reconstruction of excited state spectra **2**

4 Optimized geometries **3**

5 Application of the restricted subspace approximation **3**

1 Synthesis

Methyl 4-formylbenzoate (50 g, 0.31 mol) was added in a 1 L single necked flask and was fully dissolved in propionic acid (400 mL). The solution was heated at 100 °C and then pyrrole (20.2 mL, 0.31 mol) was added dropwise and the mixture was refluxed for 1.5 h. After the reaction completion, the mixture was cooled to room temperature and submerged in an ice bath. The precipitates were isolated through vacuum filtration, and further recrystallized and washed with methanol to obtain the pure product 5,10,15,20-tetrakis(4-methoxycarbonylphenyl)porphyrin (TCOOMePP) as a purple crystalline powder (12.2 g, 13.23 mmol, yield 17%). ^{1}H NMR spectroscopic data were similar to those previously reported in the literature.[1] [^{1}H NMR (400 MHz, $CDCl_3$) δ = 8.82 (s, 8H), 8.45 (d, J = 8.2 Hz, 8H), 8.30 (d, J = 8.2 Hz, 8H), 4.12 (s, 12H), -2.81 (s, 2H) ppm.]

TCOOMePP (11 g, 12.99 mmol) was dissolved in a (1:1) THF/MeOH mixture (400 mL) and an aqueous solution (80 mL) of KOH (44 g, 779.3 mmol) was then added. The reaction was refluxed overnight. The following day, THF and MeOH were removed under reduced pressure, water was added, and the solution was acidified with 1M HCl and the desired product was precipitated and collected though filtration. Finally, the desired product was washed with a large volume of dichloromethane and dried under vacuum to obtain 5,10,15,20-tetrakis(4-carboxyphenyl)porphyrin (TCPP) as a purple solid (10 g, 12.65 mmol, yield: 97%). ^{1}H NMR spectroscopic data were similar to those previously reported in the literature.[2] [^{1}H NMR (400 MHz, DMSO-d_6) δ = 8.86 (s, 8H), 8.38 (db, J = 7.4 Hz, 8H), 8.31 (db, J = 8.0 Hz, 8H), -2.93 (s, 2H) ppm.]

2 Full ground state and transient spectra

Figure S1 shows the ground state and transient X-ray absorption spectra of aqueous free base $TCPP^{4-}$ (pH≈12) including transitions to the anti-bonding b_{1u} (400.0 eV, 401.1 eV) and higher b_{2g}/b_{3g} vacant orbitals (402.0 eV, 403.1 eV). See the previously published $TCPP^{4-}$ (pH≈8) data[3] for details of this assignment.

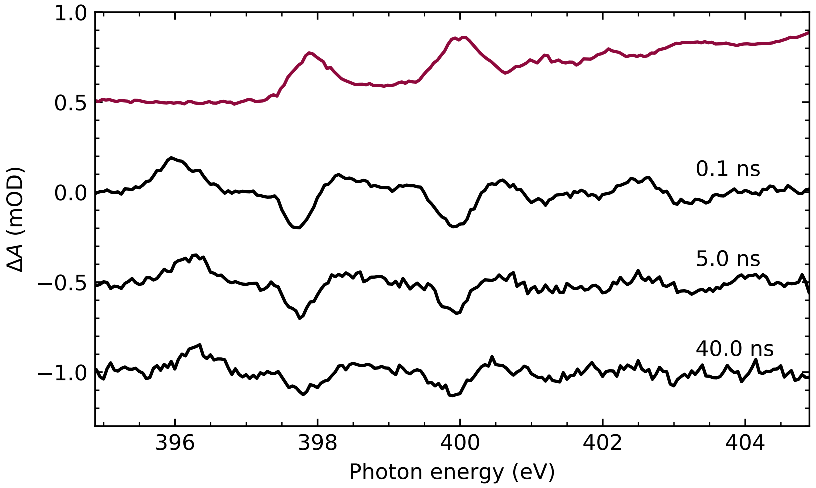

Fig. S1 $TCPP^{4-}$ N K-edge ground state (red) and transient X-ray absorption spectra 0.1 ns, 5.0 ns, 40.0 ns after 343 nm excitation (black).

3 Reconstruction of excited state spectra

The procedure to retrieve approximated N K-edge NEXAFS spectra of the lowest singlet and triplet excited states of $TCPP^{4-}$ is demonstrated in Figure S2. Compared to Figure 4a in the main text, the data is scaled here so that the shown and added ground state contributions are equal. In the main text, the resulting spectra are scaled so that the intensities of the different features is on average the same.

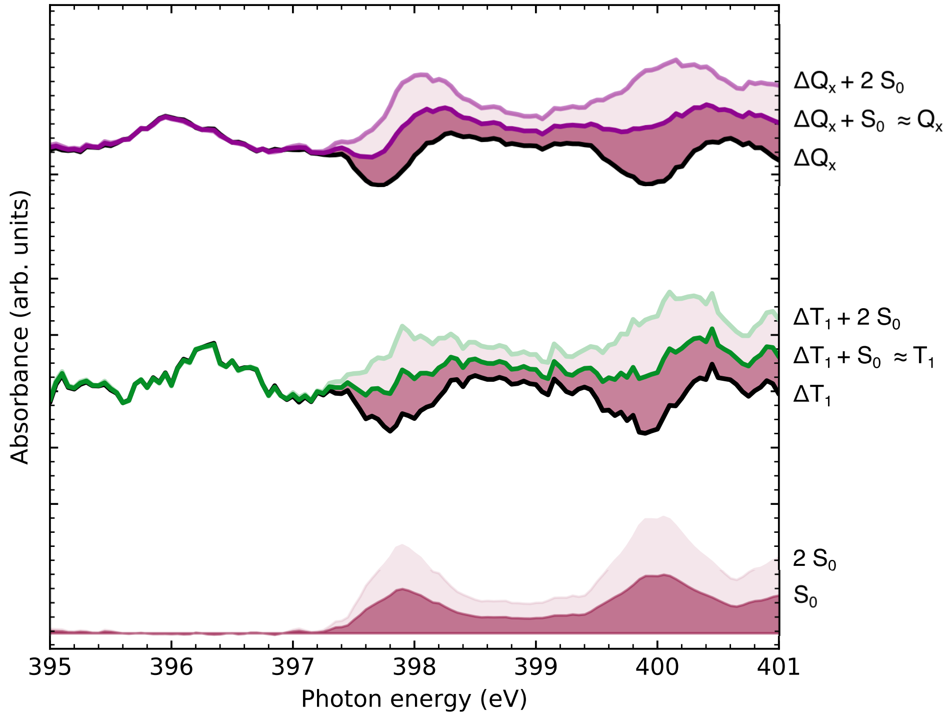

Fig. S2 Reconstruction of the T_1 (green) and Q_x (magenta) excited state spectra by adding the the ground state spectrum S_0 (red area) to the transients (ΔT_1, ΔQ_x – black). The double of the ground state spectrum 2 S_0 (light red area) has been added as well, to show that the depletion is overcompensated in this case (light green/ magenta curves).

The experimentally determined transients at pump-probe delays of 0.1 ns (ΔQ_x) and 40.0 ns (ΔT_1) are dominated by the ground state depletion above 397.5 eV. To make double excitations (as observed for a zinc porphyrin[4]) visible, the ground state spectrum (S_0)

has been added to the transient spectra. To retrieve reproducible results, the intensity of the transients has been scaled so that the absorbance at the minimum region of the depletion dips is zero.

4 Optimized geometries

The calculated out-of-plane distortions of the free base 5,10,15,20-tetraphenylporphyrin (TPP) geometry in the optically excited states (Q_x, T_1) compared to the ground state (S_0) can be seen in Figure S3.

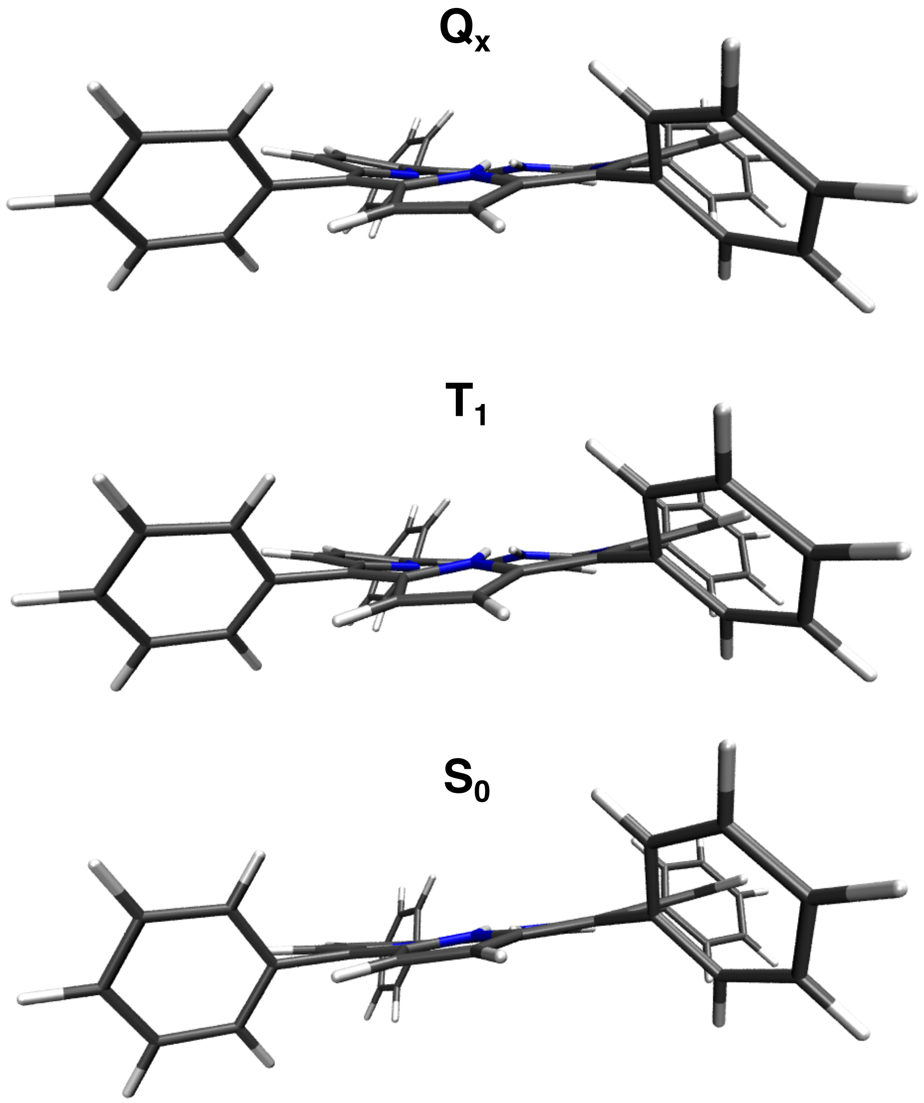

Fig. S3 Optimized geometries of TPP in the lowest singlet excited (Q_x), triplet excited(T_1), and ground state (S_0).

5 Application of the restricted subspace approximation

TD-DFT calculations within the restricted subspace approximation (RSA) were originally proposed to simulate resonant inelastic X-ray scattering (RIXS) spectra.[5] However, the procedure is also applicable to the modelling of pre-edge transient X-ray absorption features, as it has been carried out in this work. By truncating the excitation spaces we obtain the necessary valence- and core-excited states relevant to transient absorption. In this work, the virtual space was made up by the 20 lowest unoccupied orbitals, while the occupied space was comprised of the nitrogen 1s orbital (either =N− or −NH−) and the four highest occupied orbitals, leading to an orbital space (1,4,20). Then 170 roots were computed, populating the valence excitations and core excitation from the selected orbitals. The calculations were carried out from an unrestricted Kohn-Sham ground state determinant, which allows to compute also excited states with a spin flip. Lastly, the X-ray absorption spectra for each electronic state were obtained by using Multiwfn[6] to compute the transition dipole moment between the states. The ground state optical and X-ray absorption spectra of TPP are compared to the classical (full) TD-DFT outcome in Figure S4.

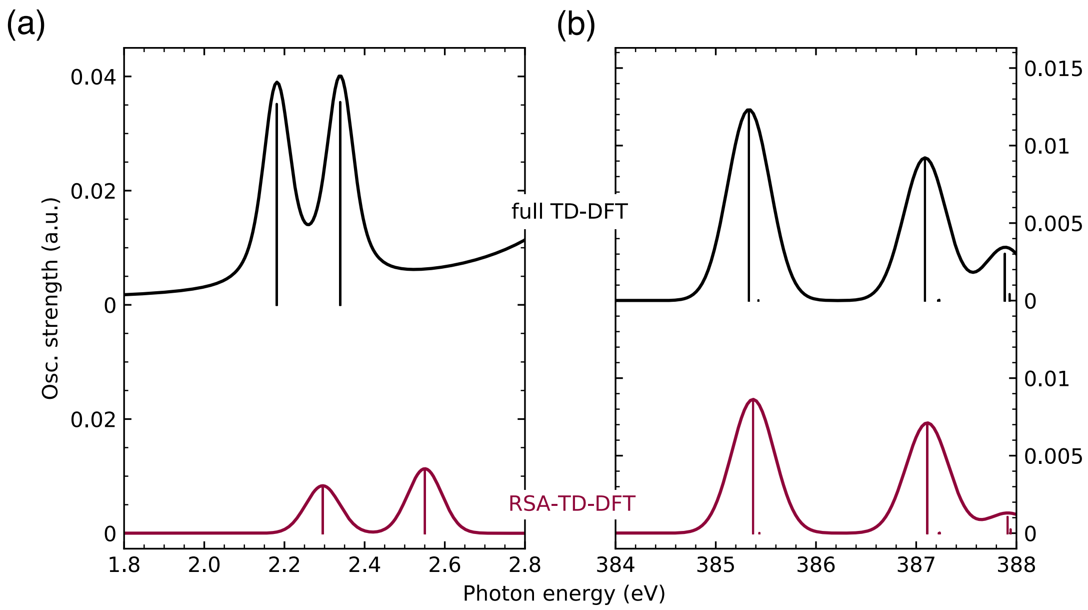

Fig. S4 TD-DFT calculation of the TPP (a) optical bands and (b) N K-edge NEXAFS within and without the restricted subspace approximation (RSA).

References

1 Y. Keum, S. Park, Y.-P. Chen and J. Park, *Angew. Chem., Int. Ed.*, 2018, **57**, 14852–14856.

2 P. Deria, J. Yu, R. P. Balaraman, J. Mashni and S. N. White, *Chem. Commun.*, 2016, **52**, 13031–13034.

3 R. Büchner, M. Fondell, R. Haverkamp, A. Pietzsch, V. Vaz da Cruz and A. Föhlisch, *Phys. Chem. Chem. Phys.*, 2021, **23**, 24765–24772.

4 A. A. Cordones, C. D. Pemmaraju, J. H. Lee, I. Zegkinoglou, M.-E. Ragoussi, F. J. Himpsel, G. de la Torre and R. W. Schoenlein, *J. Phys. Chem. Lett.*, 2021, **12**, 1182–1188.

5 V. Vaz da Cruz, S. Eckert and A. Föhlisch, *Phys. Chem. Chem. Phys.*, 2021, **23**, 1835–1848.

6 T. Lu and F. Chen, *J. Comput. Chem.*, 2012, **33**, 580–592.
